# Helping the heart grow fonder during absence: Daydreaming about significant others replenishes connectedness after induced loneliness

**DOI:** 10.1080/02699931.2015.1049516

**Published:** 2015-07-20

**Authors:** Giulia L. Poerio, Peter Totterdell, Lisa-Marie Emerson, Eleanor Miles

**Affiliations:** ^a^ Department of Psychology, University of Sheffield, Sheffield, UK; ^b^ School of Psychology, University of Sussex, Sussex, UK

**Keywords:** Daydreaming, Mind wandering, Loneliness, Belonging Regulation, Imagination

## Abstract

People are known to engage in behaviours aimed at replenishing social connectedness after their sense of belonging is threatened. We explored whether the mental strategy of daydreaming about significant others could have similar effects by acting as an imaginary substitute when loved ones are unavailable. Following a loneliness induction, participants (*N* = 126) were asked to either daydream about a significant other, daydream about a non-social scenario or complete a control task. Social daydreamers showed significantly increased feelings of connection, love and belonging compared to non-social daydreamers and control participants. Consistent with the proposition that social daydreaming replenished connectedness, social daydreamers also behaved more pro-socially and expressed less of a desire to interact with others after daydreaming. These findings demonstrate that through imagination, social daydreaming can replenish connectedness providing a potential strategy for enhancing socio-emotional well-being.

The need to form and maintain meaningful social connections is central to human experience (Baumeister & Leary, 1995). Sometimes, the need to feel interpersonally connected can be thwarted such as when dealing with rejection, ostracism or separation from loved ones. A universal form of social disconnection is loneliness: an aversive feeling accompanying the perception that the quantity or quality of social relationships are not meeting one's social needs (Russell, Peplau, & Cutrona, 1980). Loneliness can produce negative effects on cognition and behaviour and moderate levels are associated with mental health problems and poorer physical health (Cacioppo & Cacioppo, 2014).

Despite its negative associations, loneliness is proposed to be adaptive because it motivates social re-connection that would have promoted species-survival (Cacioppo, Cacioppo, & Boomsma, 2014). This is mirrored in theoretical accounts of belonging regulation, which propose that threats to belongingness motivate behaviour towards achieving a sense of interpersonal connection (Gardner, Pickett, Jefferis, & Knowles, 2005; Leary, Tambor, Terdal, & Downs, 1995). Following social disconnection, individuals engage in a range of behaviours in the service of replenishing connectedness including: behavioural mimicry to promote affiliation and rapport (Lakin, Chartrand, & Arkin, 2008), increased sensitivity to and monitoring of social information for reconnection opportunities (Gardner et al., 2005), attempts to ingratiate oneself through conforming (Williams, Cheung, & Choi, 2000) and actively seeking direct interpersonal connection (Maner, DeWall, Baumeister, & Schaller, 2007).

Although engaging in behaviours that promote social re-connection is arguably the most efficient strategy to replenish connectedness, there may be times when such behaviours are unfeasible or ineffective. For instance, loneliness might often be triggered in situations where meaningful social connection is not readily available. There is also reason to think that attempts at re-establishing social contact may hinder rather than help loneliness. This is because loneliness is associated with a cycle of negativity whereby lonely individuals hold negative social expectations about themselves and others, engage in more negative social encounters and behaviours that increase the likelihood of rejection and, as a result, may distance themselves from situations which could counteract their loneliness (Hawkley, Preacher, & Cacioppo, 2007).

How can social connectedness be replenished in situations where meaningful social contact is not readily available or may not be the optimal strategy? In this study, we test the idea that *daydreaming* about a significant other may prove an effective strategy for replenishing connectedness through imagined, rather than actual, interaction with loved ones. Daydreaming can be defined as mental content that is both unrelated to and independent of one's current task (Stawarczyk, Majerus, Maj, Van der Linden, & D’Argembeau, 2011). Although daydreaming may often occur spontaneously, such as when the mind unintentionally wanders, it can be volitional and directed when an individual chooses to initiate a particular internal thought stream and disengage from the external world (Seli, Carriere, & Smilek, 2014). We propose that such directed and volitional daydreaming may be a useful strategy to overcome loneliness because it allows the daydreamer to simulate a meaningful social interaction with a close significant other when that social contact is not available in reality. As a result, daydreaming should evoke the positive social emotions associated with the imagined event, replenishing feelings of connectedness.

Preliminary correlational evidence indicates that daydreams about close significant others may promote feelings of social connectedness. Mar, Mason, and Litvack (2012) found that self-reported daydreaming about close others was positively associated with socio-emotional well-being whereas daydreaming about non-close others was positively associated with loneliness. These associations remained after controlling for social network characteristics suggesting that daydreaming about close others may buffer against loneliness and promote social well-being. Likewise, a recent experience sampling study found that daydreams about significant others in daily life were associated with increased happiness, love and connection (Poerio, Totterdell, Emerson, & Miles, 2015). This association was observed when participants were deficient in these before their daydream suggesting that daydreaming about significant others may function to provide social sustenance through imagined, rather than actual, social contact.

## The present study

The reviewed evidence suggests that daydreaming about close others may be an effective method of replenishing connectedness. To test this idea we experimentally induced loneliness and instructed participants to either daydream about a significant other (*social daydreaming*), daydream about a pleasant, non-social event (*non-social daydreaming*) or complete a working-memory task (*control task*). We measured both negative (loneliness and social disconnection) and positive (connection with others, love, belonging) social feelings as well positive and negative affect. We hypothesised that only social daydreaming would be able to restore feelings of social connection by reducing negative and increasing positive, social feelings. This effect should be specific to social feelings rather than positive and negative affect more generally.

We provided two extensions to test the idea that social daydreams would replenish connectedness. First, we measured helping behaviour. Previous research indicates that feeling socially disconnected leads to decreased pro-social behaviour (Twenge, Baumeister, DeWall, Ciarocco, & Bartels, 2007) and that feeling socially connected leads to increased helping behaviour (Pavey, Greitemeyer, & Sparks, 2011). If social daydreaming replenishes connectedness then we would expect social daydreamers to offer to help more than non-social daydreamers and control participants. Second, we measured desire to interact with others in a subsequent task. Previous research indicates that feelings of social disconnection increase attempts to connect with others (Maner et al., 2007). If social daydreams replenish connectedness, then we would expect social daydreamers to be *less* likely to want to engage in a future interpersonal task compared to non-social daydreamers and control participants.^1^ ^1^Helping behaviour may sometimes be regarded as a form of social interaction and so might repair feelings of disconnection. However, in the present study, participants completed a helping request that was directed towards future helping behaviour and would not have involved social contact (coding data) suggesting that offers to help are less likely to be construed as a form of social interaction that might foster connectedness. We report how we determined our sample size, all data exclusions, all manipulations and all measures in the study.

## METHOD

### Participants and design

One hundred and forty-three students and staff at a UK university participated in the study for £3 (approximately $4.50). Seventeen participants were excluded from the study because they did not comply with experimental instruction (one participant in the social daydreaming condition did not describe imagining a significant other and 16 participants in the non-social daydreaming condition described social content). The final sample consisted of *N* = 126 (social daydreaming: *n* = 46, non-social daydreaming: *n* = 35, control: *n* = 45). The mean age of the sample was 23.37 years (range = 18–65, SD = 7.01) and 87 were female. Sample size was determined *a priori* with G*power3 using a medium effect size (*f* = .25), an alpha level of .05 and power at .80.

### Procedure

Participants were informed that the study was concerned with the links between imagination and cognitive abilities. All participants underwent a loneliness induction individually and were then randomly assigned to condition to complete the associated three-minute task. Participants rated their feelings three times: before and after the loneliness induction and after the experimental task. Finally, participants completed some manipulation checks, rated their desire to connect with others and completed a helping request.

### Loneliness induction

Using a procedure from Wildschut, Sedikides, Arndt, and Routledge, (2006), participants completed an ostensibly valid loneliness scale by rating their agreement or disagreement (i.e., “*agree*” or “*disagree*”) to 16 items, taken from the UCLA Loneliness Scale (Russell et al., 1980), which were worded to elicit agreement (e.g., “*I sometimes feel alone*”). Participants received bogus feedback on their level of loneliness and were told that they were in the 67th percentile of the loneliness distribution meaning they were “*much more lonely than average*”. To strengthen the manipulation participants wrote down three reasons for their score.

### Daydreaming conditions

Participants were instructed to imagine themselves in a pleasant scenario of their own choosing with the constraint that it had to be based in reality (i.e., something that had already happened or might plausibly happen in the future). To manipulate daydreaming about a significant other, social daydreamers were instructed:

What is important is that your scenario should involve interacting with another person that you have a close, positive, relationship with like a friend, family member, or a significant other. This person should be someone that you have regular contact with.

Non-social daydreamers were instructed:

What is important is that your scenario should just be about you. It shouldn’t involve thinking about or interacting with anyone else.

Participants were asked to write a sentence describing their chosen scenario, then imagine it with their eyes closed for three minutes, and write a description of what they had imagined.

### Control condition

Participants completed a three-minute 1-back working-memory task in which they responded to a stimulus only when it matched the previous stimulus. The stimuli were 12, one-syllable semantically unrelated words (*corn*, *fence*, *green*, *guard*, *jump*, *large*, *month*, *name*, *push*, *star*, *tape*, *waive*); participants pressed the space bar when the word displayed matched the preceding word which occurred 25% of the time.

### Feeling measures

Seventeen items measured current feelings. Participants rated the extent to which they felt each feeling “*right now*” from 1 (*very slightly or not at all*) to 5 (*extremely*). The order of all items was randomised for each participant each time they reported their feelings.

### *Positive and negative social feelings*

A single item measured loneliness (“*lonely*”) and three items, taken from the Social Connectedness Scale (Lee & Robbins, 1995), measured feelings of social disconnection (“*disconnected from the world around you*”, “*distant from other people*”, “*unrelated to anyone*”). These three items were averaged to create a score for social disconnection with higher values indicting greater social disconnection (average *α* = .82). Three items measured positive social feelings of connectedness (“*connected with others*”), love (“*loving*”) and belongingness (“*a sense of belonging*”).

### *Positive and negative affect*

Positive and negative affect were measured using the 10-item short form of the Positive and Negative Affect Schedule (*PANAS*; MacKinnon et al., 1999) which consisted of 10 emotion-related adjectives; five measuring negative affect (average *α* = .77) and five measuring positive affect (average *α* = .87).

### Manipulation checks

To check that participants had focused on their allocated task, they rated how much time they had spent thinking about each of the following: “*your chosen scenario/the working memory task*”, “*a close significant other*”, “*topics unrelated to the imagination/working memory task*” on scales from 1 (*none of the time*) to 5 (*all of the time*).

A Kruskal–Wallis test confirmed that social daydreamers reported spending significantly longer thinking about a close significant other (Mdn = 3) compared to non-social daydreamers (Mdn = 2, *p* < .001) and control participants (Mdn = 1, *p* < .001), *H*(2) = 36.16, *p* < .001.

Analyses also confirmed that there were no differences between conditions for time spent thinking about their allocated task, *F*(2, 123) = .59, *p* = .520, 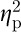 = .01 (social daydreamers: *M* = 3.59, SD = .96, non-social daydreamers: *M* = 3.83, SD = .71, control participants: *M* = 3.67, SD = 1.09) or task-unrelated thought, *F*(2, 123) = .20, *p* = .819, 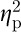 = .00 (social daydreamers: *M* = 2.37, SD = 1.02, non-social daydreamers: *M* = 2.26, SD = .92, control participants: *M* = 2.24, SD = 1.11).

Participants in the daydreaming conditions also rated the positivity of their daydream (“*The imagined scenario was*…”) from 1 (*negative*) to 5 (*positive*). Social (Mdn = 5) and non-social daydreams (Mdn = 5) were rated as equally positive, *U*(79) = 703.00, *p* = .480, *r* = .08.

### Desire to connect with others

Using a procedure from Maner et al. (2007), participants were told that another part of the study would take place either alone or with several others, and that their preference would be considered. Participants answered the question, “*To what extent would you prefer doing the next task with a few other social partners?*” from 0 (*not at all*) to 11 (*extremely*).

### Helping request

Using a procedure adapted from Vohs, Mead, and Goode (2006), participants were told that the experimenter was seeking help with coding data. They were told that each data sheet would take approximately five minutes to code, and were asked if they would be willing to help. The experimenter left the room to ostensibly prepare for the next task and participants indicated on a sign-up form how many data sheets (if any) they would code and provided their contact details.^2^ ^2^Participants who offered to code a range of sheets (e.g. 5–10) were given the mid-way point as their value (e.g. 7.5). Two participants offered to help but could not give an exact value and were excluded from analyses. Four participants offered to code a maximum number of sheets rather than specifying the number (e.g. “as many as possible”). These participants (one each in the social-daydreaming and pleasant-daydreaming conditions and two in the control condition) were given the maximum value of their condition. One participant in the social-daydreaming condition who offered to code 100 sheets was excluded from analyses as an outlier (> 2SD above the mean).


## RESULTS

### Effect of loneliness induction

After the induction, for social feelings, participants felt: lonelier (*M* = 1.72, SD = .92) than before (*M* = 1.56, SD = .84), *t*(125) = 2.09, *p* = .039, *d* = .18; more socially disconnected (*M* = 1.76, SD = .78) than before (*M* = 1.65, SD = .78), *t*(125) = 2.05, *p* = .042, *d* = .14; less connected with others (*M* = 2.80, SD = 1.07) than before (*M* = 3.09, SD = 1.09), *t*(125) = 3.83, *p* < .001, *d* = .27; marginally less loving (*M* = 2.92, SD = 1.18) than before (*M* = 3.04, SD = 1.15), *t*(125) = 1.88, *p* = .063, *d* = .10; and marginally less belonging (*M* = 2.85, SD = 1.15) than before (*M* = 2.98, SD = 1.03), *t*(125) = 1.81, *p* = .074, *d* = .12. Participants also felt less positive affect after the induction (*M* = 2.90, SD = .97) than before (*M* = 3.01, SD = .83), *t*(125) = 2.85, *p* = .005, *d* = .13, but did not feel more negative affect after the induction (*M* = 1.43, SD = .60) than before (*M* = 1.44, SD = .57), *t*(125) = .38, *p* = .702, *d* = .02, suggesting that the negative impact of the loneliness induction was isolated to social feelings rather than negative affect more generally.^3^ ^3^Feelings of loneliness, social disconnection and negative affect were all significantly positively skewed. We attempted to transform these variables but no transformation was able to adequately normalise the distribution. Although we report parametric tests for these variables for consistency, non-parametric tests produced equivalent results and are available on request. Seventeen participants expressed suspicion that the ostensibly valid loneliness scale used for the loneliness induction was not an accurate measure of loneliness. We re-ran analyses excluding these participants: results and conclusions were unaffected.


### Did social daydreams replenish connectedness?

**Figure 1:**
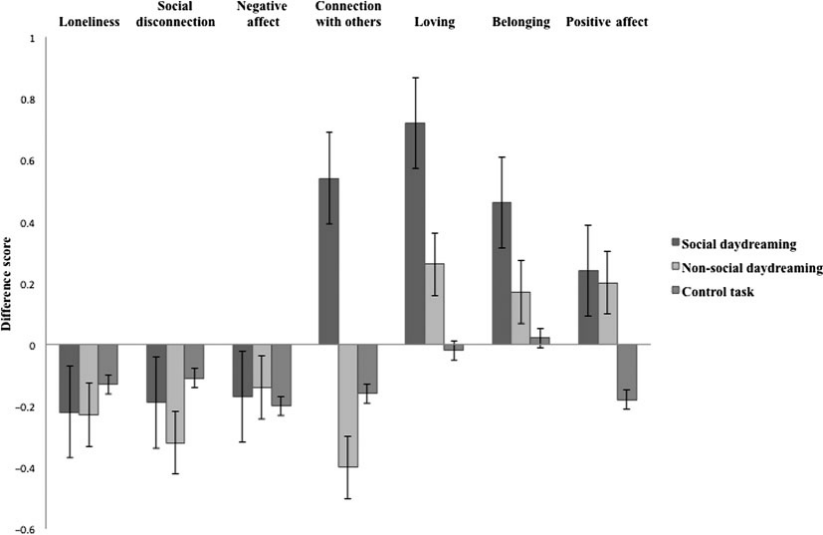
Mean difference scores (post-task feelings – pre-task feelings) as a function of condition. Error bars represent ± 1 SEM.

To test our hypothesis that social daydreams would replenish connectedness compared to non-social daydreaming or a control task, we conducted two 2-within (Time: pre-task, post-task) × 3-between (Condition: social daydreaming, non-social daydreaming, control task) MANOVAs (one for positive feelings; one for negative feelings). We were interested in significant interaction effects between time and condition, which would indicate differences in the effect of condition on positive and negative feelings before and after the experimental task. Significant interactions were followed up with a series of 2-within (Time: pre-task, post-task) × 3-between (Condition: social daydreaming, non-social daydreaming, control task) ANOVAs with each feeling state as the dependent variable, which were further investigated by comparing the simple main effects of time separately for each condition. Results are summarised in Figure 1.

For negative feelings, in contrast to hypotheses, the interaction between time and condition was non-significant, *F*(2, 123) = .63, *p* = .631, 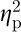 = .01. However, a significant main effect of time indicated that negative feelings decreased over time for all conditions, *F*(2, 123) = 29.73, *p* < .001, 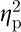 = .20. Specifically, reports of loneliness, social disconnection and negative affect decreased over time for all conditions (loneliness: *F*(1, 123) = 7.57, *p* = .007, 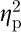 = .06 [pre-task: *M* = 1.73, SE = .08; post-task: *M* = 1.53, SE = .07]; social disconnection: *F*(1, 123) = 18.29, *p* < .001, 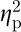 = .13 [pre-task: *M* = 1.76, SE = .07; post-task: *M* = 1.56, SE = .06]; negative affect: *F*(1, 123) = 20.47, *p* < .001, 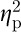 = .14 [pre-task: *M* = 1.42, SE = .05; post-task: *M* = 1.52, SE = .03]).

For positive feelings, there was a significant interaction effect between time and condition, *F*(2, 123) = 13.07, *p* < .001, 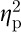 = .18. This interaction effect was observed for all positive feelings when examined separately: connection with others, *F*(2, 123) = 11.09, *p* < .001, 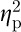 = .15; love, *F*(2, 123) = 8.38, *p* < .001, 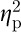 = .12; belonging, *F*(2, 123) = 3.26, *p* = .042, 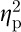 = .05; and positive affect, *F*(2, 123) = 7.22, *p* = .001, 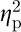 = .11. Social daydreamers felt more connected with others (*p* < .001, *d* = .50), whilst non-social daydreamers felt less connected with others (*p* = .014, *d* = .37) and control participants showed no change (*p* = .276, *d* = .14). Social daydreamers also felt more loving and belonging (*p*s < .001, *d*s = .62, .41) but non-social daydreamers (*p*s = .083, .220, *d*s = .25, .16) and control participants (*p*s = .843, .857, *d*s = .02, .02) showed no change. Both social and non-social daydreamers felt more positive affect (*p*s = .006, .042 *d*s = .23, .22) whilst control participants felt less positive affect (*p* = .035, *d* = .17) after the task.

These results suggest that social daydreaming, relative to both non-social daydreaming and the control task, increased positive social feelings of connectedness, love and belonging. Whilst both kinds of daydreaming seemed to increase positive feelings in general, only *social* daydreams were associated with increased positive social feelings.

To check that the effect of social daydreaming on social feelings held over and above the effect of positive affect more generally, we conducted a series of ANCOVAs including pre- and post-task feelings of positive affect as covariates. Interactions between time and condition remained significant for connectedness, *F*(2, 121) = 10.29, *p* < .001, 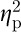 = .15 and love, *F*(2, 121) = 5.63, *p* = .005, 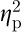 = .09, but not for feelings of belonging, *F*(2, 121) = 1.66, *p* = .195, 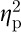 = .03. Simple effects confirmed that social daydreamers felt more connected (*p* < .001, *d* = .58) and more loving (*p* < .001, *d* = 1.05) after daydreaming, whilst non-social daydreamers felt less connected (*p* = .008, *d* = .79) and showed no change in feelings of love (*p* = .279, *d* = .36). Control participants showed no change in either feelings of connection (*p* = .520, *d* = .49) or love (*p* = .711, *d* = .06). Although the interaction for belonging was non-significant, simple effects showed that social daydreamers felt a greater sense of belonging (*p* = .001, *d* = .52) after daydreaming but non-social daydreamers and control participants showed no change (*p*s = .279, .426, *ds* = .54, .20). Overall, these results confirm that the effect of social daydreaming on increases in positive social feelings held after controlling for positive affect.

### Was social daydreaming linked with helping behaviour and the desire to connect with others?

#### Helping

A one-way between-subjects ANOVA revealed a marginally significant main effect of condition on helping, *F*(2, 121) = 2.85, *p* = .077, 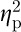 = .05. Pairwise comparisons showed that social daydreamers offered to code significantly more data sheets (*M* = 10.35, SE = 1.77) than non-social daydreamers (*M* = 4.32, SE = 2.05, *p* = .029, *d* = .44) and marginally more data sheets than control group participants (*M* = 5.73, SE = 1.81, *p* = .072, *d* = .34). Non-social daydreamers and control participants did not differ in the help they offered (*p* = .609, *d* = .19).

#### Desire to connect with others

A one-way between-subjects ANOVA revealed a marginally significant main effect of condition on desire to connect with others, *F*(2, 123) = 2.57, *p* = .081, 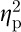 = .04. Pairwise comparisons showed that social daydreamers expressed *less* of a desire to connect with others (*M* = 4.87, SE = .41) than non-social daydreamers (*M* = 6.17, SE = .41, *p* = .037, *d* = .49) but showed no difference compared to control participants (*M* = 5.00, SE = .41, *p* = .822, *d* = .05). Control participants were also marginally less likely to want to connect with others compared to non-social daydreamers (*p* = .062, *d* = .41).

#### Supplementary mediation analysis

**Figure 2:**
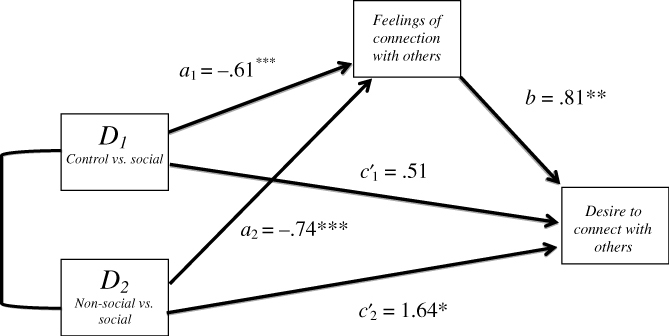
Mediation model of the effects of condition on desire to connect with others as mediated by feelings of connection with others. Social daydreaming is the reference category (coded 0), compared to the control group (D_1_) and non-social daydreaming (D_2_) (coded 1). Standardised path coefficients are shown. Total effects (c) for D_1_ and D_2_ were .02 and 1.04, respectively. Asterisks indicate significant coefficients (*p < .05, **p < .01, ***p < .001).

Given that social daydreamers felt significantly more connected and non-social daydreamers felt significantly less connected with others after daydreaming, we conjectured that feelings of connection would mediate the effects of condition on the desire to connect with others. Following Hayes and Preacher’s (2014) procedure for mediation with multi-categorical independent variables, we created two dummy variables to examine the relative effects of being in one condition (control or non-social daydreaming, coded 1) relative to a reference category (social daydreaming, coded 0), with feelings of connection before each task as a covariate in the models (results summarised in Figure 2). Post-task feelings of connectedness exerted significant indirect effects in the control, relative to social daydreaming, condition (indirect effect = −.50; 95% bootstrapped confidence interval, CI: [−1.02, −.19]) and the non-social daydreaming condition relative to the social daydreaming condition (indirect effect = −.60; 95% CI: [−1.16, −.23]). Post-task feelings of connection mediated the effect of condition on the desire to connect with others, meaning that social daydreamers expressed less of a desire to connect with others because they felt more connected after daydreaming than both non-social daydreamers and control participants.

## DISCUSSION

We tested whether daydreaming about a significant other could replenish connectedness after induced loneliness. As expected, social daydreamers showed significant increases in feelings of connection, love and belonging compared to both non-social daydreamers and control participants. Although both social and non-social daydreaming were associated with increased positive affect, only *social* daydreaming was associated with increased positive *social* feelings. Both social and non-social daydreaming were associated with increased positive affect compared to the control condition, which was associated with decreased positive affect. Daydreaming about something pleasant would therefore seem to have an emotional benefit compared to engaging in a cognitive task, presumably because of the rewarding nature of such imaginative activity. The effect of social daydreaming on positive social feelings also remained after controlling for positive affect, indicating that the observed effect occurs over and above positive affect more generally. Importantly, the effects of social daydreaming extended beyond self-reported feelings to behavioural intent, providing additional evidence that social daydreams replenished connectedness. First, social daydreamers were more helpful than non-social daydreamers and control participants, offering to code, on average, nearly twice as many data sheets. This is consistent with research linking social connection with increased helping behaviour (Pavey et al., 2011) and social disconnection with decreased pro-social behaviour (Twenge et al., 2007).^4^ ^4^We failed to find evidence that the effect of social daydreaming on helping was mediated by feelings of social connection (**results** available on request). Although we found that social daydreamers helped more than other participants, this was not due to increased positive social feelings. Second, social daydreamers expressed less of a desire to interact with others in a future task. This finding was mediated by feelings of connection; social daydreamers felt more interpersonally connected, which in turn was associated with a decreased desire for potential social connection. The decreased desire for social future interaction is what would be expected if social daydreaming had replenished participants’ sense of connectedness (Maner et al., 2007).

Although social daydreaming was uniquely linked to increase positive social feelings, it did not have the same effect on negative social feelings. Feelings of social disconnection and loneliness decreased over time for all conditions. A likely explanation for this is that participants reported only low levels of these feelings post-induction, leaving little opportunity for differential effects to occur. Indeed, a limitation of our study is that the loneliness induction produced mild levels of social disconnection and loneliness meaning that findings can only truly be applied to such mild feelings. Whether or not social daydreams are capable of reducing more intense disconnection is an open question. Future research might, therefore, use alternative methods to induce disconnection (e.g., Williams et al., 2000) or investigate social daydreaming with chronically lonely individuals.

Our findings support the proposal that social daydreams can replenish connectedness by providing an imaginary substitute for significant others when they are not immediately available. There is evidence that daydreams may function like this in daily life (Mar et al.,^ 201^2; Poerio et al., 2015) but the present study provides more direct evidence for the causal role of daydreaming about significant others in maintaining and sustaining connectedness. It is well established that the need for social sustenance drives much of human behaviour (Baumeister & Leary, 1995) to the extent that humans seek companionship from non-human agents (Epley, Akalis, Waytz, & Cacioppo, 2008). Our research suggests that going to such lengths to sustain connectedness may not be necessary. Instead, it may be achievable by helping people harness their imagination to daydream about past and possible future interactions with loved ones.

The present findings also have implications for accounts of daydreaming. Although daydreaming has often been presumed to be an idle or harmful activity, it is increasingly common to consider daydreaming as a heterogeneous phenomenon wherein effects depend on the content and context in which daydreaming occurs (Smallwood & Andrews-Hanna, 2013). The present findings contribute to this endeavour by demonstrating that volitional daydreaming about significant others (i.e., the daydream's content) in times of distress (i.e., the daydream's context) can function to replenish connectedness. Although this may be one function of daydreams about significant others, social daydreaming may serve broader functions such as anticipation, rehearsal and outcome simulation, and may also have dysfunctional elements (e.g., links with rumination and social anxiety).

We believe that our findings motivate three particularly interesting questions. First, the present study indicates that imagined interaction was a more effective strategy for replenishing connectedness than non-social daydreaming, but could imagined interaction also be more effective than actual interaction? The idea that daydreaming functions as an imaginary substitute for social interaction implies that daydreaming is in some way inferior to actual social interaction. However, there may be circumstances when imagined interaction is preferable and more effective than social interaction. Certain individuals may not feel comfortable relying on others to regulate their distress and even when significant others are available, they may not always be supportive or responsive, which may ironically exacerbate feelings of social disconnection and have deleterious interpersonal consequences (Feeney & Collins, 2003). However, imagination as a tool to foster connectedness has the advantage of being under the daydreamer's control, rather than relying on a positive response from others, allowing the daydreamer to simulate the contact they desire.

Second, what are the effects of imagined interactions on longer-term social interaction? Social daydreamers were less likely to want to interact with others because they already felt socially connected implying that this may impede the very social interaction that might buffer against longer-term social disconnection. Indeed, positive fantasies can decrease motivation and goal attainment (Kappes & Oettingen, 2011) suggesting that positive social daydreams may reduce the motivation to act and gain social sustenance from meaningful relationships. Of course, positive fantasies are not equivalent to daydreaming, and participants were instructed to imagine realistic, rather than fanciful, social scenarios rather to reflect this. However, future research could explore whether the amount or nature of social daydreaming may unduly interfere with satisfying social interaction.

Third, could social daydreaming be used for therapeutic benefit? This study demonstrates that a three-minute spell of directed daydreaming is enough to foster feelings of connectedness, love and belonging as well as promoting a prosocial orientation. Future research might explore the potential benefits of social daydreaming for individual well-being and interpersonal relationships (e.g., within the context of relationship counselling, social reintegration or social network changes). Simulating positive interactions during daydreaming may also help to target maladaptive social cognition associated with loneliness, depression and social anxiety.
